# Acute Molecular Changes in Synovial Fluid Following Human Knee Injury: Association With Early Clinical Outcomes

**DOI:** 10.1002/art.39677

**Published:** 2016-08-25

**Authors:** Fiona E. Watt, Erin Paterson, Andrew Freidin, Mark Kenny, Andrew Judge, Jeremy Saklatvala, Andy Williams, Tonia L. Vincent

**Affiliations:** ^1^ Arthritis Research UK Centre for Osteoarthritis Pathogenesis, Kennedy Institute of Rheumatology, University of OxfordOxfordUK; ^2^ Fortius Clinic, Imperial College Healthcare NHS Trust, St. Mary's HospitalLondonUK; ^3^ NIHR Musculoskeletal Biomedical Research Unit, University of Oxford, Oxford, UK, and University of SouthamptonSouthamptonUK; ^4^ Arthritis Research UK Centre for Osteoarthritis Pathogenesis, Kennedy Institute of Rheumatology, University of Oxford, Oxford, UK, and Fortius ClinicLondonUK

## Abstract

**Objective:**

To investigate whether molecules found to be up‐regulated within hours of surgical joint destabilization in the mouse are also elevated in the analogous human setting of acute knee injury, how this molecular response varies between individuals, and whether it is related to patient‐reported outcomes in the 3 months after injury.

**Methods:**

Seven candidate molecules were analyzed in blood and synovial fluid (SF) from 150 participants with recent structural knee injury at baseline (<8 weeks from injury) and in blood at 14 days and 3 months following baseline. Knee Injury and Osteoarthritis Outcome Score 4 (KOOS_4_) was obtained at baseline and 3 months. Patient and control samples were compared using Meso Scale Discovery platform assays or enzyme‐linked immunosorbent assay.

**Results:**

Six of the 7 molecules were significantly elevated in human SF immediately after injury: interleukin‐6 (IL‐6), monocyte chemotactic protein 1, matrix metalloproteinase 3 (MMP‐3), tissue inhibitor of metalloproteinases 1 (TIMP‐1), activin A, and tumor necrosis factor–stimulated gene 6 (TSG‐6). There was low‐to‐moderate correlation with blood measurements. Three of the 6 molecules were significantly associated with baseline KOOS_4_ (those with higher SF IL‐6, TIMP‐1, or TSG‐6 had lower KOOS_4_). These 3 molecules, MMP‐3, and activin A were all significantly associated with greater improvement in KOOS_4_ over 3 months, after adjustment for other relevant factors. Of these, IL‐6 alone significantly accounted for the molecular contribution to baseline KOOS_4_ and change in KOOS_4_ over 3 months.

**Conclusion:**

Our findings validate relevant human biomarkers of tissue injury identified in a mouse model. Analysis of SF rather than blood more accurately reflects this response. The response is associated with patient‐reported outcomes over this early period, with SF IL‐6 acting as a single representative marker. Longitudinal outcomes will determine if these molecules are biomarkers of subsequent disease risk.

Joint injury is a well‐established risk factor for osteoarthritis (OA) 1, 2. After meniscal tear requiring surgical intervention, ∼50% of individuals will develop OA 3, 4. Other substantial knee injuries, such as intraarticular fractures or anterior cruciate ligament (ACL) ruptures, in isolation or in combination with meniscal tears, also predispose to OA 5, 6, 7. However, there are currently no prognostic biomarkers that allow us to reliably predict which individuals with a given knee trauma will develop ongoing symptoms and/or OA. It is clear that surgical ACL reconstruction improves instability symptoms but does not remove the risk of posttraumatic OA. There is a pressing clinical need to identify the early processes in injured joints that lead to subsequent disease, and the factors that influence them. Quantifying an individual's risk of OA may allow us to intervene therapeutically in predisposed individuals.

Joint trauma is arguably an ideal experimental setting to study early or pre‐OA states. The initiating stimulus is usually temporally defined, and validated murine models are in widespread use, allowing investigation of early disease mechanisms 8. Using one such model, surgical destabilization of the medial meniscus (DMM), our group has identified the transcriptional events in the first few hours following this procedure, which reproducibly leads within weeks to OA 9. By microarray analysis and subsequent reverse transcriptase–polymerase chain reaction, many genes were found to be up‐regulated following joint‐destabilizing surgery 10. These were mostly inflammatory response genes, including cytokines such as interleukin‐6 (IL‐6), chemokines, and proteases (including metalloproteinase 3 [MMP‐3] and aggrecanases capable of initiating cartilage degradation), but also several molecules with predicted antiinflammatory or repair functions, such as activin A, a transforming growth factor β (TGFβ) family member, and tumor necrosis factor–stimulated protein 6 (TSG‐6). Both the transcriptional response to acute joint destabilization and the extent of disease development can be modulated by joint immobilization, sex, or varying genetic strain, suggesting that at least some of this immediate inflammatory gene response is necessary for, or might predict, disease development 10, 11.

Identification of biomarkers for OA has to date focused primarily on products of matrix degradation, and typically in cohorts of individuals with established OA. Although some biomarkers show statistically significant associations with severity, progression, or therapeutic response 12, 13, this field has been largely disappointing in identifying a qualified biomarker that is of use at the individual level, either in clinical trials or in practice 14, 15. Others have documented elevations of various inflammatory response molecules or their activity in synovial fluid (SF) after joint injury 16, 17, 18, 19, 20, 21, 22, 23. However, with two exceptions most of these studies were cross‐sectional and relatively small (n < 50), were of a single injury type, and lacked associated clinical data or the power to examine the effect of clinical variables or outcomes. Few studies have simultaneously measured the molecule in SF and blood and in control samples 20, 22, and no studies to our knowledge have shown associations between these measurements and validated prospective patient‐reported outcomes. To date “catabolic” or proinflammatory genes in the inflammatory response have typically been examined, rather than those thought to be antiinflammatory or promoting repair. For example, activin A and TSG‐6 have not been investigated in this context.

The Knee Injury Cohort at the Kennedy (KICK) study was undertaken to systematically test whether acute injury response molecules that were up‐regulated in the mouse joint were also elevated following human knee joint injury in a specifically designed prospective human cohort. Seven molecules were selected for investigation, from our murine studies: IL‐1β, IL‐6, monocyte chemotactic protein 1 (MCP‐1), MMP‐3, tissue inhibitor of metalloproteinases 1 (TIMP‐1), TSG‐6, and activin A 10. From a group of ∼30 up‐regulated transcripts, all 7 were highly regulated in the mouse joint; all were predicted to be secreted and therefore quantifiable in human SF, serum, or plasma; and all had assays that were validated by us to reliably measure the analyte in both SF and blood (Table 1). In this study, we investigated the presence of this biologic response in a human cohort and the association of the biologic response with clinical outcome measures over the initial months following a knee joint injury.

**Table 1:** Characterization of assays for analytes^a^

Analyte	Assay	Catalog no., manufacturer	Intraassay CV, %	Interassay CV, %	Normal range in serum, ng/ml	Normal range in plasma, pg/ml	Normal range in SF, pg/ml	SF dilution, fold
Activin A	Human activin A Quantikine kit	DAC00B, R&D Systems	2.6	6.9	–	128–403	655–5,250	50
CRP	Human vascular injury II kit	K15136C‐1, MSD	8.2	15.0	0–5,000	–	110–2,670^b^	1,000
IL‐1β	Proinflammatory 9‐plex ultra‐sensitive kit	K15007C‐1, MSD	8.0	0.8	–	<3	<3	5
IL‐6	Custom multiplex kit (IL‐6 + MCP‐1)	K15007C‐1, MSD	10.8	15.7	–	0–1.49	0–19.8	5
MCP‐1	Custom multiplex kit (IL‐6 + MCP‐1)	K151AYC‐1, MSD	6.4	21.7	–	84–499	55–487	5
MMP‐3	Human MMP 3‐plex ultra‐sensitive kit	K15034C‐1, MSD	6.2	19.9	5.3–32.0	–	0–231^b^	400
TIMP‐1	Human TIMP‐1 kit	K151JFC‐1, MSD	6.5	8.8	211–466	–	75–745^b^	200
TSG‐6	Custom human TSG‐6 prototype kit	Prototype, MSD	8.6	18.8	1.3–12.3	–	0–3.1^b^	4

a Immunoassays were performed using commercially available plate enzyme‐linked immunosorbent assays, or electrochemiluminescence (MSD). The latter included singleplex, multiplex, or prototype‐printed assays, validated by us. Plasma or serum, depending on assay, or synovial fluid (SF) aliquots were warmed to room temperature and gently vortexed prior to assay. Intraassay and interassay coefficients of variation (CV) of <12% and <25%, respectively, were established for all assays (n = 20 for intraassay CV and n = 4 or more for interassay CV). The lower and upper limits of quantitation were calculated from standard curves of 3 validation plates. Samples below the lower limit were arbitrarily assigned half the lower limit of quantitation as their concentration during analyses 22. Spike recoveries within 80% and 120% were deemed acceptable. Linearity of dilution was confirmed for all 8 assays across the dilution range used. Uninjured control samples for both fluid types were assayed and normal ranges were calculated (mean ± 2SD; n = 50 for serum/plasma and n = 8 for SF). CRP = C‐reactive protein; IL‐1β = interleukin‐1β; MCP‐1 = monocyte chemotactic protein 1; MMP‐3 = matrix metalloproteinase 3; TIMP‐1 = tissue inhibitor of metalloproteinases 1; TSG‐6 = tumor necrosis factor–stimulated gene 6.

b The unit of measure was ng/ml.

## PATIENTS AND METHODS

### Ethics approval

Approval for the KICK study was given by South East London Research Ethics Committee 5 (REC reference 10/H0805/39; NCT02667756). Written informed consent was obtained from all participants prior to screening, in accordance with the Declaration of Helsinki.

### Participants

Participants were referred for screening by the orthopedic surgeon investigator (AW) from a population with acute knee injury assessed by him at various sites in London, UK. Inclusion criteria were clinically significant acute knee injury within 8 weeks of recruitment; age 16–50 years; knee effusion, evident clinically or by magnetic resonance imaging (MRI); and evidence of ≥1 specified structural injury on MRI (Table 2). Exclusion criteria were preexisting advanced radiographic OA (Kellgren/Lawrence grade 3–4) of the injured (index) knee; inflammatory/septic arthritis of the index knee; previous or planned knee arthroplasty; active or treated systemic inflammatory disease; recent infection; pregnancy; and inability to provide blood samples. Relative exclusion criteria (at the discretion of the investigator) included bony abnormality of the index knee; injury of other body parts or surgery within the last 3 months; severe neurologic/muscle/hip disease; significant, active comorbidity; and contraindication for MRI.

**Table 2:** Characteristics of the KICK participants and controls

	KICK participants (n = 150)	Controls (blood samples)(n = 50)	Controls (SF samples)(n = 8)
Age, median (range) years	25 (16–50)^a^	32 (21–49)	48 (41–68)
Sex, no. (%) male/female	121 (81)/29 (19)	33 (65)/17 (35)	4 (50)/4 (50)
Time from injury at baseline, median (range) days	17 (1–56)	–	–
Body mass index, median (range)	26 (19–39)	Not available	Not available
Tegner score prior to injury, median (range)	10 (3–10)	–	–
Tegner score at baseline, median (range)	2 (1–6)	–	–
Type of injury, no. (%)^b^			
Meniscal tear	27 (18)	–	–
Single ligament rupture only	28 (18)	–	–
ACL + meniscal tear	61 (41)	–	–
Severe trauma	34 (23)	–	–
Clinical effusion at baseline^c^	145 (97)	–	–
SF blood staining, no. (%)^d^, ^e^			–
None	42 (31)	–	6 (75)
Mild	34 (25)	–	2 (25)
Moderate	25 (18)	–	0 (0)
Severe	26 (19)	–	0 (0)
Present, ungraded	9 (7)	–	0 (0)
KOOS_4_ at baseline, mean ± SD^e^	44 ± 18	–	–
KOOS_4_ at 3 months, mean ± SD^e^	62 ± 16	–	–
Serum CRP at baseline, median (range) ng/ml^e^	524 (26.8–56,700)	485 (43.7–5,098)	–
K/L grade at baseline, median (range)^e^	0 (0–2)	Not available	Not available

a *P* < 0.0001 versus control blood and synovial fluid (SF) samples, by Mann‐Whitney test.

b Four types of injury were defined and are listed in order of increasing extent of trauma (categorized by arthroscopy where performed, supplemented by magnetic resonance imaging [MRI] findings). Severe trauma was defined as combined ligament (>1) rupture, or fracture or dislocation. ACL = anterior cruciate ligament.

c The size of effusion was estimated clinically as small (46%), medium (39%), or large (12%). Five participants had effusions at the time of MRI that had resolved by baseline.

d The presence of blood staining in SF was graded subjectively under normal light conditions using a predefined visual grading scale, where none = no visible red staining of the SF, mild = visible red staining with a high level of translucency (finger behind tube visible with low distortion), moderate = heavy red staining with a low level of translucency (finger behind tube visible with high distortion), and severe = heavy red staining and opaque (finger behind tube not visible).

e SF blood staining data were available for 136 Knee Injury Cohort at the Kennedy (KICK) participants, Knee Injury and Osteoarthritis Outcome Score 4 (KOOS_4_) at baseline was available for 143 KICK participants, KOOS_4_ at 3 months was available for 124 KICK participants, serum C‐reactive protein (CRP) levels at baseline were available for 149 KICK participants, and Kellgren/Lawrence (K/L) grade at baseline was available for 150 KICK participants.

### Clinical outcomes

**Figure 1:**
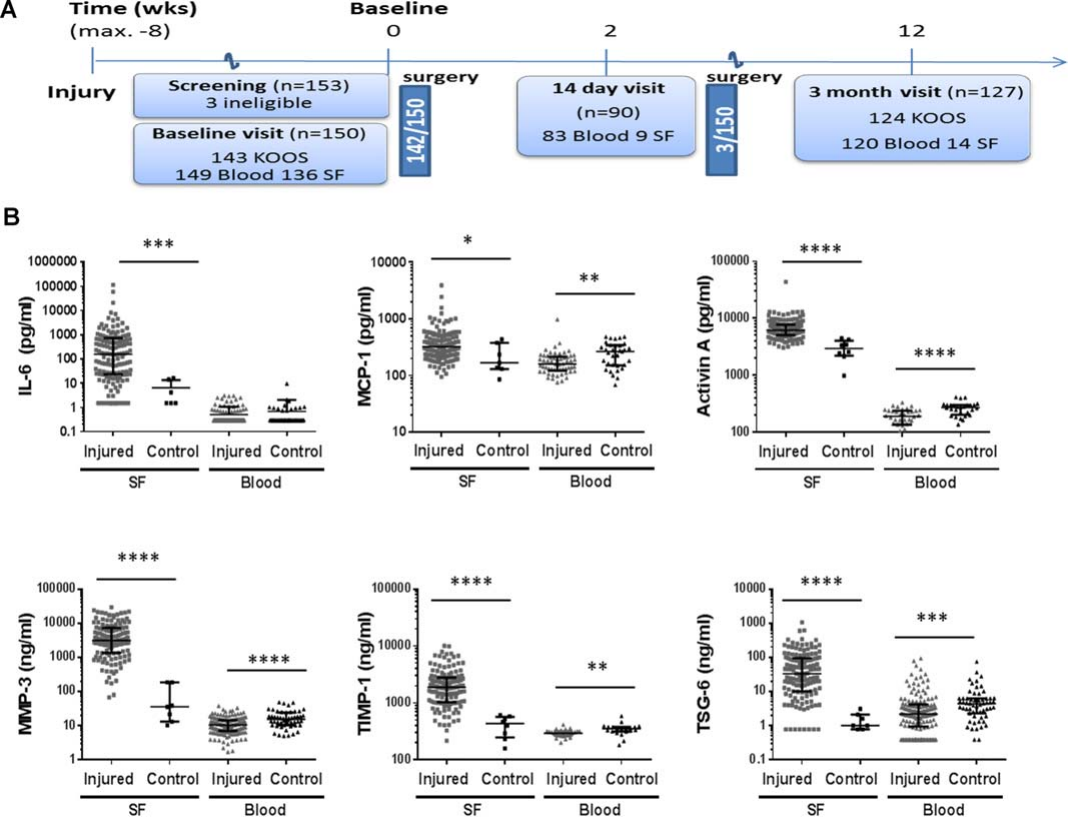
Analytes in the synovial fluid (SF) and blood of participants with knee injury in the Knee Injury Cohort at the Kennedy (KICK) and healthy controls. **A,** Flow chart indicating the schedule of study visits in the KICK cohort over 3 months and timing of clinically indicated surgical treatment. (Surgical interventions are detailed in Supplementary Table 1, http://onlinelibrary.wiley.com/doi/10.1002/art.39677/abstract.) The numbers of individuals for whom Knee Injury and Osteoarthritis Outcome Score (KOOS) data were available (at baseline and 3 months) and from whom blood and SF samples were obtained are shown. To be eligible for the study, participants had to have sustained 1 or more of the following injuries within 8 weeks of the baseline visit: meniscal tear, cruciate ligament rupture, colateral ligament tear, posterolateral corner injury, traumatic chondral defects, articular or periarticular fracture, or patellofemoral or tibiofemoral dislocation. **B,** Baseline levels of markers of interest in SF and matched blood samples, either plasma (for interleukin‐6 [IL‐6], monocyte chemotactic protein 1 [MCP‐1], and activin A) or serum (all other markers), from KICK participants with knee injury and healthy, age‐ and sex‐matched controls. All samples were centrifuged to remove cells. Supernatants were measured in duplicate by electrochemiluminescence or enzyme‐linked immunosorbent assay (activin A only). Values were obtained in all participants for all SF analytes except for IL‐6 and tumor necrosis factor–stimulated gene 6 (TSG‐6). IL‐6 levels were below the lower limit of quantitation in 12 of 136 samples (8.8%), and TSG‐6 levels were below the lower limit of quantitation in 8 of 135 samples (6%). Measurements for each of 6 analytes are shown, plotted on a log_10_ y‐axis. Symbols represent individual samples; horizontal and vertical lines show the median and interquartile range. ∗ = *P* < 0.05; ∗∗ = *P* < 0.01; ∗∗∗ = *P* < 0.001; ∗∗∗∗ = *P* < 0.0001, by Mann‐Whitney U test. MMP‐3 = matrix metalloproteinase 3; TIMP‐1 = tissue inhibitor of metalloproteinases 1. Color figure can be viewed in the online issue, which is available at http://onlinelibrary.wiley.com/journal/doi/10.1002/art.39677/abstract.

The Knee Injury and Osteoarthritis Outcome Score (KOOS), from which KOOS_4_, a single composite score, can be calculated (an average of 4 of the 5 KOOS subscales including pain, symptoms, sports/recreation, and quality of life) 24 and Tegner score 25 were obtained at baseline and 3 months (Figure 1A). Musculoskeletal examination findings, including knee effusion grade, were documented by the same investigator (FEW).

### Controls

Normal SF was obtained from patients undergoing limb amputation for treatment of lower limb tumor at RNOH Stanmore (London, UK) or transplant donation at Charing Cross Hospital (London, UK) (REC 09/H0710/60), who had macroscopically normal knee articular cartilage at the time of surgery and no evidence of arthritis or tumor invasion into the joint. Of the controls who had sarcoma, none had received chemotherapy near the time of the sample collection (within ∼6 weeks before the sample collection). Plasma and serum were obtained from consenting healthy donors who were approximately matched with the patients for age and sex (REC 11/H0711/17).

### Samples

Biologic samples were obtained at the baseline visit (within 8 weeks of knee injury and prior to any surgical intervention), and 14 days and 3 months after baseline (Figure 1A). Samples included whole blood and, where there was clinical intervention such as arthrocentesis or arthroscopy, SF. SF was collected by needle aspiration from the joint prior to introduction of the arthroscope. All samples were transferred to the laboratory within 2 hours. Whole blood (divided between tubes with EDTA and plain tubes for plasma and serum, respectively) was centrifuged at 1600*g* for 15 minutes at 20°C. SF was centrifuged for a further 20 minutes at 3,000*g*. Supernatants were stored in cryovials at −80°C in monitored freezers.

### Reagents

General laboratory reagents were the best available grade and were from either Sigma‐Aldrich or BDH unless otherwise stated. Meso Scale Discovery (MSD) plates and MSD Sulfo‐Tag–labeled streptavidin (catalog no. R32AD‐5) were from Meso Scale Discovery. Activin A Quantikine enzyme‐linked immunosorbent assay (ELISA) was from R&D Systems.

### Assays

All assays were carried out according to the manufacturers’ instructions unless stated otherwise. Each assay underwent structured validation and performance assessment for serum or plasma and for SF prior to use (Table 1). ELISA plates were read using Berthold Mithras LB940, and MSD plates were read using MSD Sector Imager 2400 and analyzed with MSD Discovery Workbench software version 3. For TSG‐6, plates were custom‐coated (MSD) with capture antibody (anti–TSG‐6) (clone NG3, MABT108; Merck Millipore), blocked in 2% bovine serum albumin (BSA)/phosphate buffered saline (PBS), and washed 3 times with PBS/0.05% Tween 20. Twenty‐five microliters per well of standards (250‐0.24 ng/ml of recombinant human TSG‐6) (#2104‐TS‐050; R&D Systems) or samples (in Cusabio sample diluent [MBS926793; MyBioSource]) were incubated in duplicate for 2 hours. Sulfo‐Tag–labeled streptavidin (1 μl/ml) was added to detection antibody (human TSG‐6 biotinylated polyclonal antibody [0.5 μg/ml]) (BAF2104; R&D Systems). Plates were washed, and 25 μl of detection solution was added per well for 2 hours. Plates were washed and incubated with 150 μl/well of read buffer (MSD).

### Data storage and statistical analysis

Power calculations to allow detection of a change in outcome of KOOS_4_, applying a minimum clinically important difference of 8 and SD of 15, were carried out at the initiation of the study 24, based on a 2‐sided *t*‐test comparing high and low levels of a particular biomarker. Fifty‐six individuals in each group (total population of 112) were adequate to give 80% power at a 5% level of significance. Allowing for ∼30% dropout, 150 individuals were recruited.

#### Comparison of biomarker levels between KICK participants and controls

Normality of each continuous variable was assessed by Q–Q plots and kernel density distribution plots. For nonparametric variables, differences between 2 groups were compared by Mann‐Whitney U test.

#### Change in biomarker levels over time (baseline, 14 days, and 3 months)

For comparison of more than 2 groups of normally distributed variables, repeated‐measures analysis of variance was used.

#### Association of biomarkers with KOOS_4_

The outcome measures were KOOS_4_ at baseline and absolute change in KOOS_4_ between baseline and 3 months. Linear regression was used to model the relationship between biomarker levels and the outcome variable KOOS_4_, adjusting for 7 predefined confounding variables (time from injury to sampling, extent/type of joint injury, SF blood staining, presence of effusion, sex, age, and body mass index [BMI]). Crude unadjusted models describe the association of the biomarkers with the outcome, and adjusted models were controlled for 4 significant explanatory variables (time from injury to sampling, extent/type of joint injury, heavy SF blood staining, and age).

All available data from all participants from relevant visits were analyzed. Data were stored in a secure online database (System for Collaborative Translational Research [Hospital for Special Surgery]). Analysis was performed in Stata IC 13 (StataCorp) and GraphPad Prism 6.03.

## RESULTS

### Characteristics of the study population

Characteristics of the 150 individuals in the cohort are shown in Table 2. The group was young and predominantly male (only 29 of the 150 participants were female). The participants’ median Tegner score prior to injury was 10, indicating a high level of physical activity (10 equates to national elite competitive sports). Of the 150 participants, 71% were professional sports players. The majority (95%; n = 143) had sustained their injury playing sports, 76% of these playing either football or rugby. Other modes of injury included tripping, and skiing or ballet injuries.

Participants were recruited soon after knee injury, with a median time from injury to baseline visit of 17 days (Figure 1A). At baseline, the majority of participants had moderate to severe pain in their index knee (>4 on a scale of 0–10, with 10 indicating the worst pain imaginable), and the KOOS_4_ showed significant impairment (where 100 is normal and 0 is highly impaired) (Table 2). All subjects had evidence of clinical effusion at baseline, or had prior evidence of effusion on MRI. There were a range of structural knee injuries within the cohort; these were categorized via arthroscopic findings (subsequently performed for clinical reasons), supplemented by MRI findings. Injury categories are listed in Table 2 in order of increasing extent of trauma; injuries tended to be structurally more extensive as the category increased. The most common findings were a meniscal tear, an ACL rupture, or both. A total of 145 participants underwent surgical treatment of their injury, 140 of these <24 hours after the baseline visit (see Supplementary Table 1, available on the *Arthritis & Rheumatology* web site at http://onlinelibrary.wiley.com/doi/10.1002/art.39677/abstract). SF was available for 136 (91%) of the participants at baseline; some blood staining was seen in the majority of these samples (Table 2). Data and sample completeness at other visits are shown in Figure 1A. There was 1 case of septic arthritis at 6 weeks, and this participant was withdrawn from subsequent study follow‐up and from the analysis at this point.

### Elevated levels of candidate molecules in SF, but not peripheral blood, after knee injury

Levels of 6 of the 7 analytes, IL‐6, MCP‐1, activin A, MMP‐3, TIMP‐1 and TSG‐6, were significantly elevated in the SF of KICK participants, compared with SF samples from 8 controls (Figure 1B). However, values varied widely between individuals, with a substantial number lying within the normal range for some molecules, such as IL‐6 and TSG‐6. IL‐1β levels were below the limit of detection for the assay for all but 1 individual (data not shown).

In contrast, there was no significant elevation of the analytes in the blood of KICK participants at baseline (although some individuals had elevated IL‐6 or TSG‐6 levels) (Figure 1B). Paradoxically, all analytes except for IL‐6 were significantly lower in blood from the KICK participants than in the 50 controls.

### The longitudinal response to joint injury is detectable in peripheral blood for some molecules, but correlates poorly with that in SF

**Figure 2:**
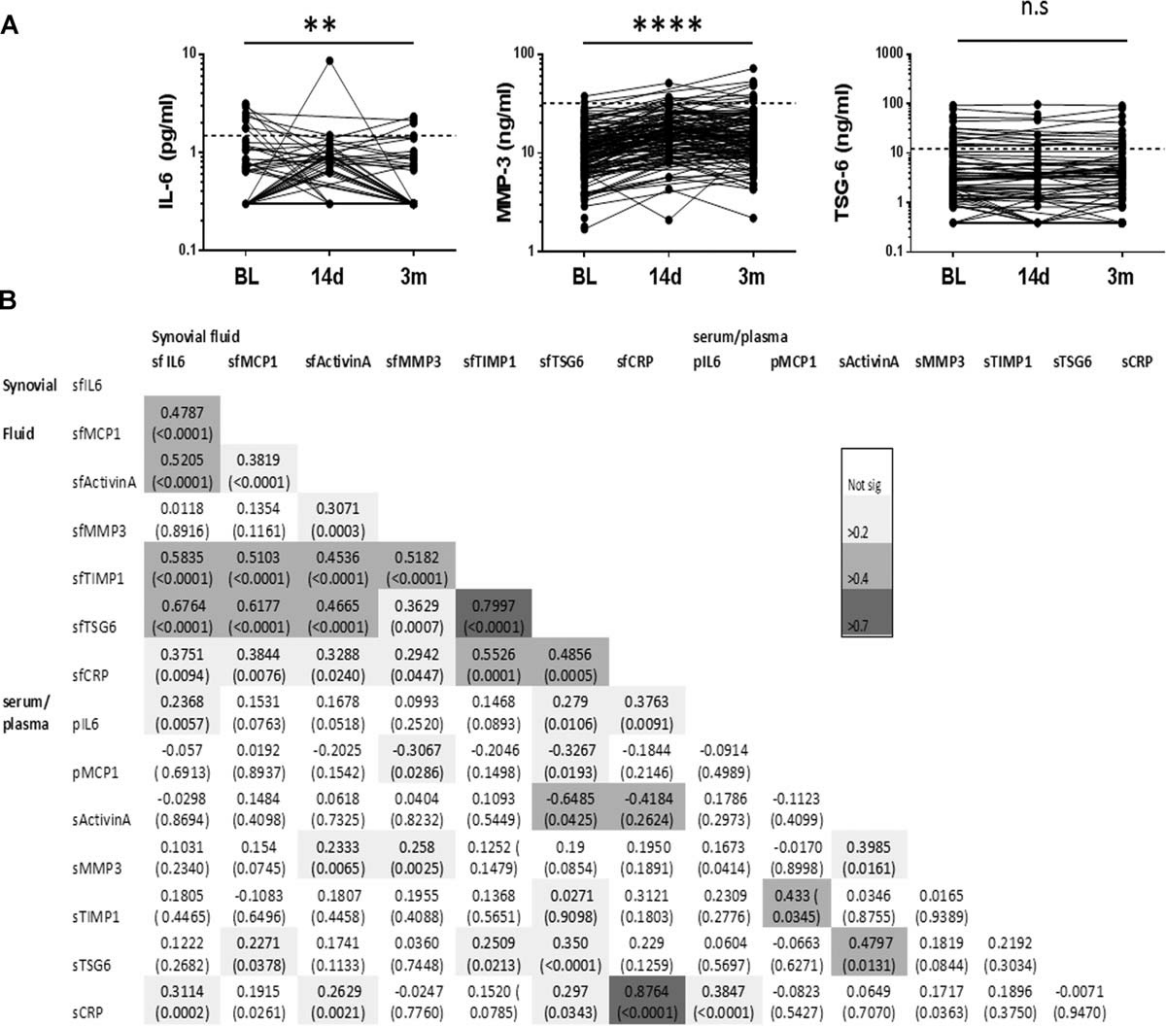
Change in analyte levels over time in the blood of KICK participants, and correlation between analyte levels in SF and blood. **A,** Blood samples, either plasma (IL‐6) or serum (MMP‐3 and TSG‐6), from individuals with knee injury were obtained at the baseline (BL) visit (within 8 weeks of injury), and 14 days and 3 months after the baseline visit and assayed for IL‐6, MMP‐3, and TSG‐6. Values for each individual are connected by a line. IL‐6 levels were below the lower limit of quantitation in 120 of 149 samples at baseline, 58 of 82 samples on day 14, and 104 of 120 samples at 3 months. TSG‐6 levels were below the lower limit of quantitation in 32 of 149 samples at baseline, 17 of 53 samples on day 14, and 19 of 120 samples at 3 months. Broken lines represent the calculated upper limit of normal for each analyte. The significance of the difference over time for each log‐transformed analyte level was tested by repeated‐measures analysis of variance. ∗∗ = *P* < 0.01; ∗∗∗∗ = *P* < 0.0001. NS = not significant. **B,** Correlations between analyte levels in SF, serum (s), and plasma (p), were determined by performing nonparametric Spearman's rank test on nontransformed data. All available participant data from the baseline visit were analyzed. Values are Spearman's R coefficient with *P* values in parentheses. Shading indicates the strength of correlation. CRP = C‐reactive protein (see Figure 1 for other definitions).

Given the low detectable response in the blood samples at baseline, it was important to assess if there was delayed response in serum or plasma at the 3‐month visit. IL‐6 levels were very low or undetectable; for those with measurable levels at baseline there was a tendency for IL‐6 levels to decrease, whereas serum MMP‐3 levels increased compared with baseline levels (Figure 2A). However, serum MMP‐3 levels were above the upper limit of normal at 3 months for only a few individuals. All individuals who had elevated serum TSG‐6 levels at baseline continued to have significantly elevated serum levels at 3 months. A group within the control population who had relatively higher serum levels of TSG‐6 was also evident (Figure 1B). MCP‐1, activin A, and TIMP‐1 levels were low and there was very little detectable change in blood over 3 months (data not shown).

There was moderate‐to‐strong correlation between the levels of several SF analytes in samples at baseline, notably IL‐6, TIMP‐1, and TSG‐6 (Figure 2B). In contrast, there was at best low‐to‐moderate correlation between paired plasma or serum and SF levels for any given analyte, except for C‐reactive protein (CRP) (Spearman's R = 0.88, *P* < 0.0001) (Figure 2B). CRP levels were not significantly elevated in the SF or serum of those with joint injury compared with controls, although a few individuals did have elevated serum levels at baseline (Table 2). CRP levels were substantially lower in SF than in serum, likely reflecting generation from a systemic source rather than the joint. In view of the lack of any satisfactory surrogate blood marker(s) for the immediate response to joint injury, it was important to focus on the large and detectable response in the SF.

### The variation of the injury response is partly explained by time from, and severity of, injury

**Figure 3:**
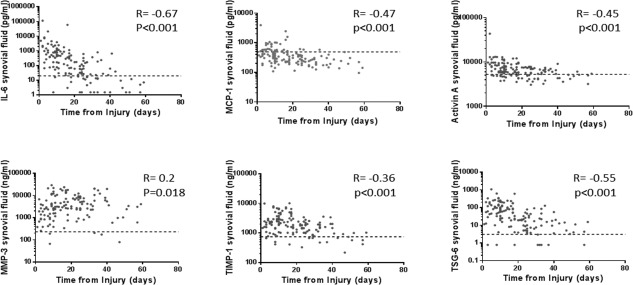
Influence of time from injury on the biologic response to injury in SF. Measurements of the levels of 6 analytes in SF obtained from the index knee at the baseline visit were plotted on a log_10_ y‐axis against time from injury to sampling (within 8 weeks of injury). Nontransformed data are shown for all analytes. Symbols represent individual samples. Broken lines represent the calculated upper limit of normal for each analyte. Spearman's R coefficient and *P* values are shown. See Figure 1 for definitions.

It was likely that levels of SF markers would be higher the closer the sampling was to the time of joint injury. This was true for all analytes. Samples obtained within 20 days of injury had the highest levels (Figure 3). MMP‐3 levels appeared least affected by time from injury to sampling. Nontransformed data are shown in Figure 3. After log transformation of marker levels, a linear relationship with time from injury accounted for 15–30% of the variation in markers.

Substantial interindividual variation in these SF markers was therefore not accounted for. Log‐transformed SF analyte levels were modeled by linear regression with 7 predefined explanatory variables: time from injury, type/extent of joint injury, presence of SF blood staining, presence of large effusion, sex, age, and BMI (Table 2 and Supplementary Table 2, available on the *Arthritis & Rheumatology* web site at http://onlinelibrary.wiley.com/doi/10.1002/art.39677/abstract). Increasing extent of knee trauma was significantly associated with increases in most analytes. The presence of hemarthrosis (moderate or severely blood‐stained SF) and age were also significant explanatory variables for some analytes. No significant independent effect on any of the analytes was found for sex, BMI, or size of effusion.

### Independent association between the molecular response to injury in SF and KOOS_4_

**Figure 4:**
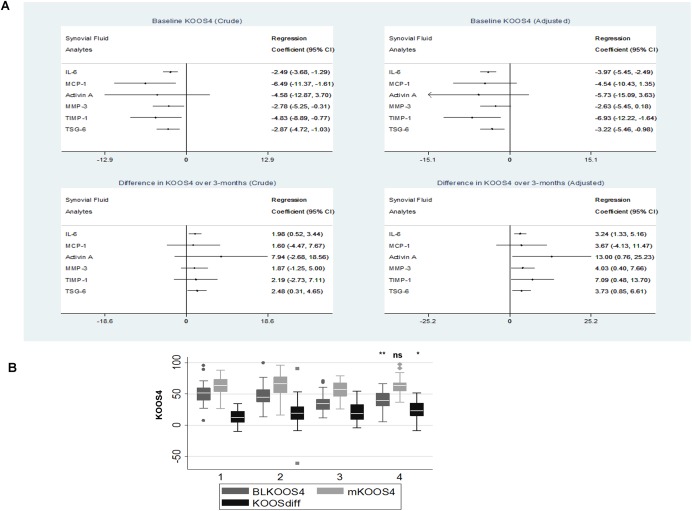
Association of SF analyte levels, including IL‐6 levels, with the clinical outcome KOOS_4._ **A,** Linear regression models of participants’ KOOS_4_ at baseline (top) and of the change in KOOS_4_ over 3 months (bottom) for each of 6 SF analytes at baseline. Forest plots of unadjusted (crude) results are shown on the left, and forest plots of results adjusted for 4 predefined variables (time from injury, injury category, presence of SF blood staining, and age) are shown on the right. 95% CI = 95% confidence interval. **B,** KOOS_4_ at baseline (BLKOOS4), KOOS_4_ at 3 months (mKOOS4), and change in KOOS_4_ over 3 months (KOOSdiff) for KICK participants grouped into quartiles of SF IL‐6 levels at baseline, with group 1 being the lowest quartile and group 4 being the highest quartile. Data are shown as box plots. Each box represents the 25th to 75th percentiles. Lines inside the boxes represent the median. Lines outside the boxes represent the 10th and 90th percentiles. Symbols indicate outliers. ∗ = *P* < 0.05; ∗∗ = *P* < 0.01 versus group 1 (lowest quartile of IL‐6 levels), by Mann‐Whitney U test. NS = not significant (see Figure 1 for other definitions). Color figure can be viewed in the online issue, which is available at http://onlinelibrary.wiley.com/journal/doi/10.1002/art.39677/abstract.

SF IL‐6, TIMP‐1, and TSG‐6 levels were each significantly associated with baseline KOOS_4_ (Figure 4A). When all 3 markers were added to a linear regression model of baseline KOOS_4_, IL‐6 alone was significant in representing the molecular response in SF, alongside blood staining and time from injury (coefficient −4.0 [95% confidence interval (95% CI) −5.92, −2.08]) (adjusted R^2^ for model 0.30, contribution of 3 analytes 0.16).

The association of each SF analyte with change in KOOS_4_ over 3 months was investigated, adjusting for relevant explanatory variables. In the absence of analyte measurements, only 2% of the difference in KOOS_4_ over the 3 months was explained (injury category was the only significant variable). Baseline SF IL‐6, activin A, MMP‐3, TIMP‐1, and TSG‐6 levels were each significantly associated with the change in KOOS_4_ over 3 months, whereas MCP‐1 was not (Figure 4A). IL‐6 levels were most strongly associated with the change in KOOS_4_. When all 5 of these SF analytes were included in the linear regression model, only SF IL‐6 levels were independently associated with the change in KOOS_4_ (coefficient 2.80 [95% CI 0.89, 5.52]).

Participants with SF IL‐6 levels in the highest quartile had significantly lower (worse) KOOS_4_ at baseline compared with individuals with IL‐6 levels in the lowest quartile, whereas these 2 groups had a similar KOOS_4_ at 3 months. On average, KOOS_4_ increased by 10 points more over 3 months in those with IL‐6 levels in the highest quartile at baseline compared to those with IL‐6 levels in the lowest quartile at baseline (Figure 4B). The differences between those with low IL‐6 levels and those with high IL‐6 levels held true if participants were divided into subgroups based on the severity of injury. Scores for the KOOS domains pain, symptoms, and sports and physical activity were all significantly reduced (worse) at baseline in those with high SF IL‐6 levels (*P* = 0.0002, *P* = 0.0035, and *P* = 0.04, respectively), and the quality of life domain score was reduced to a lesser extent (*P* = 0.07).

Findings were the same if the 4 individuals from whom SF was obtained 14 days after baseline were included (data shown do not include these individuals). A post hoc analysis of individuals who reported oral nonsteroidal antiinflammatory drug (NSAID) use within 10 days of either their baseline or 3‐month visit (49 individuals), and those who did not (101 individuals) suggested that NSAID use had no significant effect on baseline KOOS_4_ (*P* = 0.90), 3‐month KOOS_4_ (*P* = 0.60), or change in KOOS_4_ (*P* = 0.595), or on levels of SF IL‐6 (*P* = 0.45).

## DISCUSSION

In a large cohort of participants, nearly all of whom had paired SF and blood samples available at baseline, we observed markedly elevated SF levels of 6 of 7 molecules that were candidates identified in a mouse model of joint destabilization 10. These findings demonstrate the commonality between the mouse and human response to joint injury and support the utility of such preclinical models to investigate pathways in early OA.

To our knowledge, this is the largest study of its type, systematically examining SF and blood levels of candidate markers across a variety of knee injuries with longitudinal follow‐up. Although the levels of markers increased with the extent of injury, the ubiquitous nature of the response irrespective of the type of injury was notable. Similar increases in IL‐6 levels have been reported after isolated ACL tears, ACL tears associated with meniscal injury, and low‐ or high‐energy intraarticular fractures 22, 26, 27. It may be that the type of joint injury, rather than its cause, is more important, pointing to an underlying common pathologic response to connective tissue injury. The finding that hemarthrosis independently influences biomarker levels is consistent with previously published results 21. Nontraumatic hemarthrosis has historically been associated with arthritis and may be proinflammatory 28, 29. This is an important consideration as clinical practice moves away from early drainage or washout of joints to more conservative approaches. Our study highlights the importance of having a large enough cohort to adjust for such factors.

Notably, very little of the SF response was reflected in the paired blood samples at baseline. Our findings are consistent with recent publications from the Knee Anterior Cruciate Ligament, Nonsurgical versus Surgical Treatment (KANON) cohort, which showed levels of SF IL‐6 at baseline similar to those in the present study and increased markers of proteolysis in the SF of 121 individuals with ACL rupture, which also correlated poorly with levels in blood over a 5‐year period 22. A further smaller study conducted within 4 weeks of knee injury demonstrated that of 7 biomarkers whose levels were significantly higher in SF than in serum, there was correlation with serum levels for just 4 20. In recent years there has been a focus on serum and urine biomarkers for OA detection and prognosis 14, 15, 30, 31. While identification of a reliable marker that is detectable in more accessible biologic samples remains an important goal, ours and other studies examining both SF and blood highlight the potential loss in sensitivity if only blood is examined. It may be that in a more chronic setting after injury, the predictive value of some blood‐based markers improves 17.

This is the first study to identify TSG‐6 and activin A as highly regulated molecules in SF following human joint injury. Activin A plays a role in repair and wound healing 32, 33. We have previously shown that it is actively synthesized by cartilage in response to experimental injury, and that it exerts an anticatabolic effect on IL‐1–induced aggrecan degradation in vitro 34. TSG‐6 forms complexes with hyaluronan and inter‐α–inhibitor (IαI) 35, 36. It reportedly protects against inflammatory arthritis by reducing cartilage and bone turnover 37. Genome‐wide linkage analyses associate TSG‐6 with OA 38. In OA, those with an elevated TSG‐6:IαI ratio have a higher risk of progression to total joint replacement 39. The magnitude of response of either molecule following joint injury could influence an individual's propensity or otherwise to OA. Findings for the other 4 molecules are consistent with results of previous cross‐sectional studies in those with joint injury, showing elevated SF levels of IL‐6 and MCP‐1 18, 23, MMP‐3 17, 40, 41, and TIMP‐1 16, 19. Maximum levels of SF IL‐1β are observed within 24 hours after ACL rupture 18. The longer median time to sampling of 17 days may explain the lack of detectable IL‐1 in the present study.

IL‐6, TIMP‐1, and TSG‐6 levels were each associated with the KOOS_4_ clinical score at baseline and with the change in KOOS_4_ over a 3‐month period. SF IL‐6 accounted for this independently, making any additional contribution of TIMP‐1 or TSG‐6 redundant. It is important to interpret these findings cautiously. That higher measurable levels of inflammation, represented here by SF IL‐6, are associated with worse clinical symptoms, such as pain and loss of function, is perhaps not surprising. However, it is noteworthy that measurement of this single marker could, independently of other factors, account for as much as 16% of interindividual clinical variation. Sometimes simple clinical measurements provide the same information as biomarkers, but in this study measures such as presence of effusion were inferior to SF IL‐6. Although those with high SF IL‐6 levels were more impaired at baseline, they reached a similar point by 3 months to those with low IL‐6 levels, suggesting that the presence of inflammation after any given injury is not an early adverse prognostic factor (at least following surgical management of the injury). It is possible that a greater inflammatory response may predict a greater associated reparative response by the individual, or simply that there is more room for improvement for these individuals in this timeframe.

The IL‐6 response to injury in the joint may be biologically important. IL‐6 is synthesized by chondrocytes and synoviocytes, has the potential to initiate joint damage, and can sensitize joint nociceptive C‐fibers 42. It is often elevated in SF samples from patients with established OA and rheumatoid arthritis, and is a therapeutic target for the latter 43. However, genetic deletion of IL‐6 has no impact on, or may even worsen, murine OA 44, 45. Alternatively, it may be that the IL‐6 response is no more important than the up‐regulation of these other molecules, but its measurement most accurately represents the overall molecular response, or elements of this. SF IL‐6 showed the highest correlation with other analytes (including those associated with repair), the greatest up‐regulation after injury, and also the greatest variation between individuals. Similar features of the IL‐6 response were noted after ACL rupture 22. Interestingly, average pain score after meniscal injury was associated with increased IL‐6, MCP‐1, MIP‐1β, or interferon‐γ levels in lavaged SF, but no prospective data or validated patient‐reported outcomes were collected 23. In those undergoing partial meniscectomy, histologic synovial inflammation was associated with worse preoperative symptoms, but not with poorer outcomes in the first 2 years after arthroscopy 46, which supports our findings.

Our study has some limitations. We showed that examination of SF appears to have more utility than examination of blood, but we were not able to systematically collect interval SF samples. Only 18 individuals (with ongoing clinical problems) were resampled, so analysis of this nonrepresentative subgroup was not included. Systematic longitudinal collection of SF samples is clearly desirable when possible. The 93% rate of SF sampling in this cohort was possible because the vast majority of participants underwent planned early surgical interventions. Because of this, it is impossible to distinguish between the longitudinal response to injury and the response to surgical intervention. It will be important to examine whether the same is found in a conservatively managed cohort.

Another limitation includes the relatively small number of SF samples from controls, and the significantly different age distributions of controls compared with individuals with joint injury. Consideration of “normal” biomarker levels should be tempered by this. Controls with high levels of athletic activity would have been preferable. The unexpected elevations in levels of analytes such as MCP‐1 in the blood of (less active) healthy controls may reflect an “immunosuppressive” role of high levels of athletic activity 47. Interestingly, reduced levels of serum markers following trauma compared with pretrauma levels were observed in a young, athletic population 48.

This study has demonstrated a quantifiable cellular response to joint injury, best represented by measurement of SF levels of IL‐6, which varies between individuals and is associated with clinical symptoms measured by KOOS_4_ in the early period after injury. It will be important to investigate whether any of this early molecular response can predict clinical or radiologic outcomes in the years after knee injury.

## AUTHOR CONTRIBUTIONS

All authors were involved in drafting the article or revising it critically for important intellectual content, and all authors approved the final version to be published. Dr. Watt had full access to all of the data in the study and takes responsibility for the integrity of the data and the accuracy of the data analysis.

### Study conception and design

Watt, Saklatvala, Williams, Vincent.

### Acquisition of data

Watt, Paterson, Freidin, Kenny, Williams.

### Analysis and interpretation of data

Watt, Paterson, Freidin, Judge, Vincent.

## Supporting information

Supplementary Table 1. KICK participants undergoing surgical interventions of the index knee between baseline visit and 3 month visit
Supplementary Table 2. Linear regression of analyte levels with significant explanatory variables.
