# Supplementary material for: Acute Molecular Changes in Synovial Fluid Following Human Knee Injury: Association With Early Clinical Outcomes
Source: Arthritis Rheumatol. 2016 Aug 25;68(9):2129–40. doi: 10.1002/art.39677 (PMC5006850; doi:10.1002/art.39677)
Supplement: Supplementary file 1 — Supplementary Table 1. KICK participants undergoing surgical interventions of the index knee between baseline visit and 3 month visit Supplementary Table 2. Linear regression of analyte levels with significant explanatory variables. [file ART-68-2129-s001.doc]

Supplementary Table 1. KICK participants undergoing surgical interventions of the index knee between baseline visit and 3 month visit

| **Type of surgical intervention** | **Number (%)** |
| --- | --- |
| ***Meniscal surgery (partial resection/repair)***  ***Single ligament repair or reconstruction only*** | 26 (17)  29 (19) |
| ***ACL reconstruction and meniscal surgery (partial resection/repair)***  ***Combined (>1) ligament reconstruction***  ***Patellar stabilisation/fracture stabilisation/chondral microfracture only*** | 57 (38)  22 (15)  10 (7) |
| ***No surgery*** | 6 (4)* |
| ***TOTAL*** | **150 (100)** |

The numbers and percentages undergoing different types of surgical intervention for their knee injury within the first 3 months of the study are given, as detailed in Figure 1A. Categories have been selected to be directly related to the injury categories given in Table 2. 144/150 underwent primary surgical treatment of their injury. Of the 6 patients who did not undergo surgery during this period, 2 participants had meniscal tears, 1 had a posterolateral corner sprain, 2 had MCL tears and 1 had an isolated complete ACL rupture (reconstructed subsequently at 8 months). All were managed with appropriate bracing and physiotherapy.

The median time from baseline visit to surgical procedure was <1 day. Of the 5 participants who underwent later surgical intervention within the 3 months, the intervention was within 2 and 6 weeks of baseline.

Of those undergoing single ligament surgery, the majority were ACL reconstructions. Of those undergoing ACL reconstruction, all either hamstring or patellar autografts and some individuals underwent a lateral tenodesis in addition.

4 individuals required a second episode of surgery to the index knee during the first 3 months: 2 required manipulation under anaesthetic only, and 2 required arthroscopic debridement.

**Supplementary Table 2.** Linear regression of analyte levels with significant explanatory variables.

| **Synovial Fluid Analyte** | **Regression coeff. (CI) for explanatory variable** | | | | |  |  | **Adj. R-squared** |
| --- | --- | --- | --- | --- | --- | --- | --- | --- |
|  | Age | Log Time from injury | Blood staining | Injury category | | BMI | Gender |  |
| **IL-6** | -0.01(-0.05, 0.035) | -1.79 (-2.23, -1.35) | 0.39 (-1.11, 0.27) | | 0.85 (-0.18, 1.88) | -0.006 (-0.083, 0.72) | -0.55 (-1.58, 0.47) | 0.45 |
| **MCP-1** | 0.0007  (-0.012, 0.013) | -0.27  (-0.40, -0.15) | 0.40 (0.20, 0.60) | | 0.18 (-0.15, 0.51) | -0.005 (-0.03, 0.17) | 0.026 (-0.26, 0.32) | 0.31 |
| **Activin A** | 0.005  (-0.003, 0.013) | -0.26  (-0.33, -0.18) | -0.07 (-0.09, 0.054) | | 0.30 (0.12, 0.48) | 0.006 (-0.008, 0.020) | -0.12 (-0.29, 0.063) | 0.30 |
| **MMP-3** | -0.027 (-0.05, -0.001) | 0.24 (0.007, 0.49) | 0.32 (0.08, 0.71) | | 1.42 (0.90, 2.08) | 0.03 (-0.013, 0.08) | -0.078 (-0.66, 0.50) | 0.27 |
| **TIMP-1** | -0.016 (-0.03, -0.002) | -0.30 (-0.43, -0.16) | 0.28 (0.06, 0.50) | | 0.99 (0.67, 1.31) | -0.009 (-0.033, 0.016) | -0.069 (-0.38, 0.25) | 0.43 |
| **TSG-6** | -0.019 (-0.05, 0.013) | -0.78 (-1.09, -0.48) | 0.79 (0.30, 1.28) | | 1.14 (0.42, 1.85) | 0.0072 (-0.47, 0.061) | -0.59 (-1.29, 0.12) | 0.38 |

Linear regression modelling is shown for synovial fluid analytes. Log transformation of synovial fluid analyte levels and time from injury was carried out, to normalise the data for this purpose. The model includes each of 6 synovial fluid analytes, adjusted for 7 pre-defined variables (Age, Log Time from Injury, Blood Staining of synovial fluid, Injury Category/type of injury (see Table 2), BMI and Gender. (Size of effusion at baseline was also included initially but did not contribute significantly for any analyte in addition to these other variables, and substantially weakened the model so is not included here). Regression coefficient (coeff.) and confidence interval (CI) are shown for each variable. The 4 variables which reached significance for any analyte were Age, Log Time from Injury, Blood Staining of synovial fluid, Injury Category. An adjusted (adj.) R-squared value for each model including the analyte level and all significant explanatory variables is shown.
